# Metabolomic signatures of the steroid biosynthesis driving sex differences in clinical asthma subtypes

**DOI:** 10.21203/rs.3.rs-9696323/v1

**Published:** 2026-06-04

**Authors:** Gbenga Dairo, Rinku Sharma, Rachel S. Kelly, Kevin Mendez, Michael J McGeachie, Scott T. Weiss, Jessica Lasky-Su, Priyadarshini Kachroo

**Affiliations:** 1 Institute of Quantitative Biomedicine, Rutgers-The State University of New Jersey, New Brunswick, United States; 2 Department of Health Informatics, Rutgers School of Health Professions, Rutgers-The State University of New Jersey, Newark, United States; 3 Channing Division of Network Medicine, Department of Medicine, Brigham and Women’s Hospital and Harvard Medical School, Boston, Massachusetts, United States

**Keywords:** asthma, subtype, metabolomics, sex, precision medicine, inhaled corticosteroids

## Abstract

**Background:**

Asthma is a heterogeneous disease with well-documented sex differences in prevalence. However, the sex-specific and pubertal effects of inhaled corticosteroids on endogenous steroid metabolites and their relationship with asthma-related clinical outcomes remain poorly understood.

**Methods:**

Targeted whole-blood plasma profiling of the steroid biosynthesis pathway was generated by Precion Inc. (NC, USA). Sixteen metabolites from the steroid biosynthesis pathway were quantified at baseline and end-of-trial in children with asthma from the Childhood Asthma Management Program (CAMP; n=1,041), aged 5–12 years. Linear mixed models and generalized linear models were used to evaluate sex-specific longitudinal and endpoint responses of steroid metabolites and clinical outcomes with inhaled corticosteroid (ICS) use, adjusting for age, sex, race, height, and body mass index.

**Results:**

ICS use in males was associated with significantly lower cortisol and cortisone levels at Year 4 (end of trial). In females, cortisol and cortisone showed more stable trajectories with ICS use, which resulted in a non-significant reduction at year 4. Males receiving ICS showed suggestive positive longitudinal associations for 17α-hydroxyprogesterone, estrone, and testosterone, while females showed a suggestive positive trajectory for androstenedione. ICS exposure modified the associations between metabolites and clinical outcomes (FEV1, FVC, eosinophils, and airway hyperresponsiveness) in a pathway- and sex-specific manner. Overall, ICS attenuated longitudinal associations between endogenous steroids and clinical outcomes, particularly in females, while selected relationships from the androgen–lung function associations were preserved at the end of the trial.

**Conclusion:**

We identify sex-specific differences in ICS-associated metabolite profiles and clinical outcomes, supporting the need for further investigation into sex-informed approaches to corticosteroid use in pediatric asthma.

**Trial registration:**

Clinicaltrials.gov: NCT00000575; registered 1999-10-27

## Introduction

Asthma is a chronic inflammatory respiratory disorder that significantly impacts the quality of life of over 24 million individuals in the United States; however, there is a disparity in its prevalence across sexes and age groups (1). The age-related male-to-female prevalence ratio in asthma has been shown to shift from 63:37% in the 2–13 age group to 53:47% in the 14–22 age range and further to 35:65% in individuals aged 23–64 (2). Similarly, a significant increase in female asthma prevalence in the 20–29 and 50–59 age groups compared with males has been demonstrated (1). Females’ lungs demonstrate higher height-adjusted forced expiratory flow rates than males, and the ratios of forced expiratory volume in one second (FEV1) to forced vital capacity (FVC) are higher in females than in males (3, 4). Also, boys and young men have higher atopy and IgE levels compared to girls and young women (5, 6), which may partially explain the higher asthma prevalence in males than females at a young age (7). However, as females reach the reproductive (14–49 yrs) and menopausal periods (> 50 yrs), their asthma rates significantly increase (7). Also, estrogen and progesterone have been reported to be implicated in asthma pathogenesis (8). On the other hand, increased levels of androgens, including testosterone or dehydroepiandrosterone (DHEA), have been associated with decreased asthma risk in males (9), providing further evidence of airway responsiveness to sex hormones.

Steroids, such as inhaled corticosteroids (ICS), are effective treatments for asthma, and have been shown to significantly decrease the likelihood of hospital admissions in both adults and children experiencing acute asthma, particularly during severe exacerbations (10). However, exogenous corticosteroid use has been linked to suppression of the hypothalamic-pituitary-adrenal (HPA) axis, leading to adrenal insufficiency (11). Despite the critical importance of the role of steroids, sex, and medication use in asthma during puberty has not been extensively studied. Furthermore, the potential for oral and inhaled steroids to bias the metabolite associations due to correlation with severity and steroid treatment further complicates the overall understanding of the steroid pathway metabolites in asthma.

The literature highlights distinct genetic and environmental contributions to asthma heterogeneity that vary across sex, lung function, inflammation, allergy, airway hyperresponsiveness, and treatment responses. An improved understanding of these relationships would be critical to dissecting patterns of asthma severity in both sexes over puberty. While the multi-ome can explain different molecular drivers of asthma pathophysiology, metabolites or small molecules have been used extensively as disease biomarkers due to their ability to most closely reflect both environmental influences and underlying genetic precursors for a wide range of health outcomes (12). Despite this progress, many of these studies are underpowered and lack appropriate study design or validation in independent cohorts and fail to capture the disease heterogeneity. Several metabolomic studies have been performed to date and have identified key metabolic pathways associated with asthma, including sphingolipids, steroids, and other microbial-related metabolites (13–17). Even in some of the large-scale cohort studies, the number of asthma cases is often limited. Furthermore, technologies such as nuclear magnetic resonance (NMR) fail to capture some of the most important metabolic pathways in asthma, particularly steroid metabolism. Therefore, as a follow-up to global metabolomics, it is essential to conduct targeted analyses to obtain a high-resolution view of specific metabolic pathways of interest and to gain deep insights into the most relevant biomarkers that could demonstrate the therapeutic potential of metabolomics (17). In our prior large-scale global metabolomics study (>14,000 participants), we identified and replicated key metabolites from the steroid biosynthesis pathway that drive the risk of adrenal suppression in patients on long-term ICS use (13). As a follow-up, in this study, we carefully selected and quantified 16 metabolites from the steroid biosynthesis pathway using targeted metabolomics in plasma from 1,041 children enrolled in the large longitudinal pediatric asthma clinical trial of ICS use, the Childhood Asthma Management Program (CAMP). We evaluated the sex-specific effect of ICS intake on steroid metabolite levels and identified associations with several asthma-related clinical outcomes, including airway hyperresponsiveness (AHR), pulmonary lung function measures (FEV1 and FVC), and Th2 inflammation markers (eosinophil counts). Our findings highlight the clinical utility of carefully monitoring specific metabolites in relation to ICS intake and enhance our understanding of shifts/trends in asthma severity between sexes.

## Methods

### Study Cohort: The Childhood Asthma Management Program (CAMP)

CAMP (Clinicaltrials.gov: NCT00000575; registered 1999-10-27) is a multi-center, randomized, double-masked clinical trial of low-dose inhaled corticosteroid use, including 1,041 children aged 5–12 years with mild-to-moderate asthma. Detailed study design and subject recruitment procedures have been previously described (18–20). Institutional review board approval was obtained at all participating centers, and informed consent/assent was obtained for all participants. For this study, we included participants with available plasma metabolomics data at both baseline and end of the trial at year 4 (N=664).

### Metabolomic Profiling: Targeted Assay data for Steroid biosynthesis

A separate batch of serum samples (at baseline and endpoint) from the same subjects was sent to Precion Inc. for absolute quantification of a comprehensive steroid hormone panel, including 16 endogenous steroids in the following sub-pathways: androgens, estrogens, progestogens, glucocorticoids, and mineralocorticoids. The Comprehensive Steroid Hormone Panel utilizes 200 μl of serum or plasma, calibrated across analytes. An analysis is conducted via three LC-MS/MS methods: Method 1 for aldosterone, estrone, and estradiol in ESI negative mode; Method 2 for non-conjugated steroids, including prednisone and prednisolone in ESI positive mode; and Method 3 for steroid sulfates in ESI negative mode. Each method employs stable labeled internal standards for quantitation through linear regression analysis, with calibration and quality control (QC) standards maintaining accuracy within ±15% of the nominal value and precision ≤10%.

## Statistical Analyses

### Longitudinal Analysis of ICS Effects on Steroid Metabolites

We used linear mixed-effect models (LMMs) to investigate the associations between ICS exposure and longitudinal changes in steroid metabolites over 4-years. Models included fixed effects for treatment group (ICS vs. non-ICS), time (baseline vs. Year 4), and their interaction, with subject-specific random intercepts to account for repeated measures. For each steroid metabolite, we fitted the following primary model:

$${\text{Y}}_{\text{ij}}=\beta_{0}+\beta_{1}ICS_{i}+\beta_{2}{\text{Time}}_{ij}+\beta_{3}\left(ICS_{i}\times {\text{Time}}_{ij}\right)+\sum_{k=1}^{p}\alpha_{k}X_{ik}+u_{i}+\epsilon_{ij}$$

Where:

Y_ij_= metabolite level for subject *i* at time *j* (*baseline or year* 4).
*ICS*_*i*_ = treatment group indicator (0 = *Non – ICS* (*Nedocromil + placebo*): *Reference*), 1 = *ICS*(*budesonide*).
*Time*_*ij*_ = time point indicator (*baseline* (*Reference*) *or year* 4)
*X*_*ik*_ = covariate *k* (age, sex, race, BMI)
*α*_*k*_ = coefficient for covariate *k*
$u_{i}\sim \text{N}\left(0,\sigma_{\text{u}}^{2}\right)$ = random intercept for subject.
$\epsilon_{ij}\sim \text{N}\left(0,\sigma_{\text{e}}^{2}\right)$ = residual error.

In this model, the main effect (β_1_) represent the difference in metabolite levels between the ICS and non-ICS groups at baseline. Because both groups were not exposed to ICS at baseline (pre-intervention), this parameter does not represent a treatment effect; instead, it captures residual baseline differences between groups. The primary parameter of interest was the treatment × time interaction (β_3_), representing the differential change in metabolite levels over time between groups. Models were adjusted for age, sex, race, and BMI. A positive β_3_ indicates that ICS users experienced less decline or greater increase in the metabolite over 4 years relative to non-users, whereas a negative β_3_ indicates greater decline or less increase.

As a sensitivity analysis, we additionally fitted baseline-adjusted mixed models that included baseline metabolite levels as covariates. The results are shown in the Supplementary Materials.

Models were fitted using restricted maximum likelihood (REML) via the lme4 package (version 1.1.37) in R. Hypothesis tests for fixed effects used Wald tests based on the ratio of coefficient estimates to their standard errors. Confidence intervals were obtained using the profile likelihood method via the confint() function.

### Endpoint Analysis of ICS Effects on Steroid Metabolites

As a follow-up analysis, we performed a cross-sectional comparison of Year 4 metabolite levels using analysis of covariance (ANCOVA), which estimates the difference in metabolite levels between ICS and non-ICS groups at the study endpoint while adjusting for baseline metabolite levels. For each steroid metabolite, we fitted the following linear regression model using only Year 4 (endpoint) data:

$$Y_{i,\text{Year}\;4}=\beta_{0}+\beta_{1}ICS_{i}+\beta_{2}Y_{i,\text{baseline}}+\sum_{k=1}^{p}\alpha_{k}X_{ik}+\epsilon_{i}$$

Where:

*Y*_*i*,*Year* 4_ = metabolite level for subject *i* at Year 4
*ICS*_*i*_ = treatment group indicator (0 = Non – ICS(Nedocromil + placebo): Reference, 1 = ICS (budenoside).
*Y*_*i,baseline*_ = baseline metabolite level for subject *i*.
*X*_*ik*_ = covariate *k* (age, sex, race, BMI).
*α*_*k*_= coefficient of covariate *k*
$\epsilon_{i}\sim \text{N}\left(0,\sigma_{\text{e}}^{2}\right)$ = residual error.

Here, the coefficient β_1_ represents the difference in metabolite levels between the ICS and non-ICS groups at Year 4, after adjusting for baseline metabolite levels and covariates. A negative β_1_ indicates lower metabolite levels in ICS users at the endpoint, whereas a positive β_1_ indicates higher levels. The coefficient β_2_ represents the baseline-endpoint slope, quantifying how well baseline metabolite levels predict year 4 levels. LMMs and ANCOVA models address complementary questions: longitudinal change versus adjusted endpoint differences.

Given known sex differences in steroid metabolism during childhood, and potential sex-specific responses to exogenous glucocorticoid exposure, we next examined whether ICS–metabolite associations differed by sex and pubertal stage. Pubertal stage was approximated using age-at-randomization-based cutoffs, and individuals in transitional age ranges were excluded to minimize misclassification (details in the supplement). Within each sex–puberty stratum, longitudinal LMMs and endpoint ANCOVA models were fitted as described above.

### Homogeneity of Slopes Assumption Test

The relationship between baseline and endpoint metabolite levels is assumed to be the same across treatment groups in standard ANCOVA. To verify this assumption and to identify potential baseline-dependent treatment effects, we tested for an ICS × baseline metabolite interaction by fitting an extended model:

$$Y_{i,\text{Year}\;4}=\beta_{0}+\beta_{1}ICS_{i}+\beta_{2}Y_{i,\text{baseline}}+\beta_{3}\left(ICS_{i}\times Y_{i,\text{baseline}}\right)+\sum_{k=1}^{p}\alpha_{k}X_{ik}+\epsilon_{i}$$

The interaction term (β3) tests whether the baseline-endpoint slope differs between treatment groups. A significant interaction (p < 0.05) indicates heterogeneous slopes, meaning that the strength of baseline-Year 4 tracking differs by treatment status. We concluded that slopes were homogeneous when the interaction was not significant (p > 0.05), thereby satisfying the ANCOVA assumption, and proceeded with the standard model. For metabolites with significant interactions, we retained the interaction term and examined baseline-endpoint slopes within each treatment group separately to characterize how ICS modified metabolic tracking. The slope patterns reveal whether ICS treatment maintains or diminishes inter-individual metabolic variability.

Models were fitted using ordinary least squares (OLS) via the lm() function in R. Hypothesis tests for the ICS effect used t-tests based on the ratio of coefficient estimates to their standard errors. Confidence intervals were obtained using the standard Wald method via the confint() function.

### Longitudinal Analysis of Metabolite - Clinical Outcome Associations

To examine whether steroid metabolite levels are associated with clinical outcomes (lung function measures (FEV1 and FVC), immune phenotypes (eosinophil counts), and airway hyperresponsiveness), and whether these associations differ by ICS treatment status, we fitted linear mixed-effects models stratified by ICS group and sex. For each metabolite–clinical outcome pair, separate models were fitted within the non-ICS and ICS strata:

$${\text{Y}}_{\text{ij}}=\beta_{0}+\beta_{1}M_{ij}+\beta_{2}{\text{Time}}_{ij}+\beta_{3}\left(M_{ij}\times {\text{Time}}_{ij}\right)+\beta_{4}M_{i,\text{baseline}}+\sum_{k=1}^{p}\alpha_{k}X_{ik}+u_{i}+\epsilon_{ij}$$

Where:

Y_ij_= clinical outcome (FEV1, FVC, LNPC20, EOS) for subject *i* at time *j*.
*M*_*ij*_ = metabolite level for subject *i* at time *j*.
*Time*_*ij*_ = time point indicator (*baseline* (*Reference*) *or year* 4).
*M*_*i,baseline*_ = baseline metabolite level for subject *i*.
*X*_*ik*_ = covariate *k* (age, height, race, BMI).
*α*_*k*_= coefficient of covariate *k*
$u_{i}\sim \text{N}\left(0,\sigma_{\text{u}}^{2}\right)$ = subject-specific random intercept.
$\epsilon_{ij}\sim \text{N}\left(0,\sigma_{\text{e}}^{2}\right)$ = residual error.

Within each ICS stratum, main effect (β_1_) represents the cross-sectional association between metabolite level and clinical outcome at baseline, conditional on baseline metabolite level and covariates. Metabolite × time interaction (β_3_) represents the change in the metabolite–outcome association from baseline to Year 4. Hence, β_3_ quantifies whether the relationship between metabolite levels and clinical outcomes evolves differently over time within each ICS stratum. We compared β_1_ and β_3_ estimates between non-ICS and ICS strata to assess whether ICS treatment modifies metabolite–outcome associations. Differences in interaction estimates across strata indicate that the longitudinal association between metabolites and clinical outcomes varies by ICS exposure.

### Endpoint Metabolite - Clinical Outcome Association Study

To assess associations between steroid metabolites and clinical outcomes at Year 4, and to determine whether these associations differ by ICS treatment status, we performed multivariable linear regression stratified by ICS group (non-ICS vs ICS) and sex. For each metabolite–clinical outcome pair, separate linear models were fitted within each stratum using only Year-4 observations:

$$Y_{i,\text{Year}\;4}=\beta_{0}+\beta_{1}M_{i,\text{Year}4}+\beta_{2}M_{i,\text{baseline}}+\sum_{k=1}^{p}\alpha_{k}X_{ik}+\epsilon_{i}$$

Where:

*Y*_*i,Year* 4_ = clinical outcome (FEV1, FVC, LNPC20, EOS) for subject *i* at Year 4
*M*_*i,Year*4_ = metabolite level for subject *i* at year 4.
*M*_*i,baseline*_ = baseline metabolite level for subject *i*.
*X*_*ik*_ = covariate *k* (age, height, race, BMI).
*α*_*k*_= coefficient of covariate *k*
$\epsilon_{i}\sim \text{N}\left(0,\sigma_{\text{e}}^{2}\right)$ = residual error.

Here, β_1_represents the Year-4 metabolite–outcome association, adjusted for baseline metabolite level and covariates (i.e., the association between *M*_*year*4_ and *Y*_*year*4_ among individuals with similar baseline metabolite values), and β_2_ captures the contribution of baseline metabolite to the Year-4 outcome (baseline adjustment term), reflecting persistence of baseline metabolite differences into Year 4. We compared β_1_ estimates between the non-ICS and ICS strata to evaluate whether ICS exposure modifies metabolite–outcome associations at Year 4. Differences in β_1_ across strata indicate that the relationship between metabolites and clinical outcomes at Year 4 differs depending on ICS treatment status.

Models were fitted using ordinary least squares (OLS) linear regression (lm) in R. Statistical inference for regression coefficients was based on t-tests, and confidence intervals were computed using the *confint*() function. We considered p < 0.05 as statistically significant and p < 0.10 as suggestive in all analyses.

All metabolite concentrations were natural log-transformed and Pareto-scaled before analysis. Regression coefficients are therefore reported in Pareto-scaled units. All analyses were conducted in R version 4.5.1 (21). The full reproducible code is available in the supplementary materials

## Results

### ICS Exposure and Steroid Metabolite Levels in the Full Cohort

**Figure 1.**
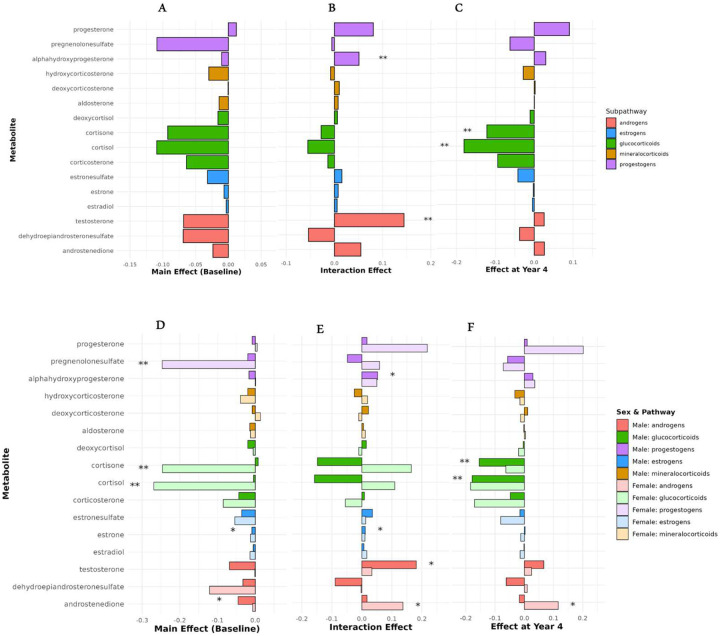
Longitudinal and endpoint associations between ICS exposure and circulating steroid metabolites in children with asthma. (A–C) Results from the combined cohort analysis. (A) Main effect (i.e., effect at preintervention) of ICS exposure on circulating steroid metabolite levels estimated from a linear mixed model without adjusting for metabolite at preintervention. (B) Interaction effect between ICS use and time (baseline to Year 4), representing differential metabolite trajectories between ICS users and non-users without adjusting for baseline metabolite. (C) Endpoint associations at Year 4 estimated using ANCOVA models. (D–F) Sex-stratified analyses showing the corresponding effects in males and females. (D) Main effect of ICS exposure. (E) ICS × time interaction representing differential longitudinal changes. (F) Endpoint associations at Year 4 from ANCOVA models. ** indicates p < 0.05; * indicates p < 0.10.

Clinical characteristics at baseline and end of the trial are summarized in Table 1. At baseline, metabolites in the glucocorticoid and androgen sub-pathway, as well as pregnenolone sulfate from the progestogen sub-pathway, were lower in all participants randomized to ICS compared to non-ICS users (Figure 1A). After baseline adjustment, the greater decline in these metabolites persisted in direction, except for DHEAS (**Supplementary Figure 1A**). In longitudinal analyses, ICS exposure was associated with directional decreases in cortisol and cortisone over the 4-year follow-up; however, the treatment × time interaction was not statistically significant (Figure 1B). In contrast, endpoint analyses showed significantly lower cortisol (Figure 1C; β = −0.18, 95% CI: −0.32 to −0.04, p = 0.01) and cortisone (β = −0.12, 95% CI: −0.22 to −0.01, p = 0.03) concentrations at Year 4 in the ICS group. Two metabolites, including 17α-hydroxyprogesterone and testosterone, demonstrated significant longitudinal differences by treatment (Figure 1B; 17α-hydroxyprogesterone: β = 0.05, 95% CI: 0.01 to 0.10, p = 0.03; Testosterone: β = 0.14, 95% CI: 0.01 to 0.28, p = 0.04), indicating relative increases over time in ICS-treated participants compared with non-users. These differences stayed robust even after baseline adjustment (**Supplementary Figure 1B**). However, they were not statistically significant at Year 4, although the direction of effect remained consistent (Figure 1C).

### Sex-Specific Effects of ICS on Steroid Metabolite Change and Endpoint Levels

At baseline, females randomized to ICS had significantly lower levels of pregnenolone sulfate, cortisol, and cortisone compared to those randomized to non-ICS (Figure 1D), a pattern less pronounced in males. In longitudinal analyses, females receiving ICS showed directionally stable, modest increases in cortisol and cortisone, whereas males showed directionally stable decreases. However, these effects were not statistically significant (Figure 1E). In baseline-adjusted longitudinal sensitivity analyses, males receiving ICS showed a suggestive trend for lower cortisone levels (**Supplementary Figure 1D**; cortisone: β = −0.14, 95% CI: −0.31 to 0.02, p = 0.09). In endpoint analyses, ICS use in males was associated with significantly lower cortisol and cortisone levels at Year 4 (Figure 1F; cortisol: β = −0.18, 95% CI: −0.35 to −0.01, p = 0.04; cortisone: β = −0.15, 95% CI: −0.29 to −0.02, p = 0.03). In females, cortisol and cortisone levels were also lower in ICS users at Year 4; however, these differences did not reach statistical significance (Figure 1F). For other metabolites, males receiving ICS showed suggestive positive longitudinal associations for 17α-hydroxyprogesterone (Figure 1E; β = 0.05, 95% CI: 0.004 to 0.11, p = 0.07), estrone (Figure 1E; β = 0.01, 95% CI: −0.002 to 0.02, p < 0.09) and testosterone (Figure 1E; β = 0.18, 95% CI: −0.01 to 0.37, p = 0.06), while females showed a suggestive positive trajectory for androstenedione (Figure. 1E; β = 0.14, 95% CI: −0.003 to 0.28, p = 0.053). These associations were attenuated at the endpoint in males, but the direction of effect remained positive and suggestive for androstenedione in females (Figure 1F; β = 0.12, 95% CI: −0.02 to 0.25, p = 0.09). Notably, 17α-hydroxyprogesterone and testosterone maintained a positive effect and significance in males, and androstenedione maintained a positive association that was significant in females in the baseline-adjusted sensitivity analyses (**Supplementary Figure 1D**).

Stratification by pubertal stage and sex was limited by small sample sizes in several strata (**Supplementary Table 1**). In prepubertal males, no significant associations between ICS and longitudinal metabolite trajectories or endpoint levels were observed (**Supplemental Figure 2A-E**). In post-pubertal females (**Supplementary Figure 3A-E**), ICS exposure was associated with increased cortisone over time in unadjusted (**Supplementary Figure 3B**; β = 0.38, 95% CI: −0.05 to 0.82, p = 0.09) and adjusted models (**Supplementary Figure 3D**; β = 0.39, 95% CI: 0.03 to 0.74, p = 0.04). Further, we observed suggestive reductions in cortisol (**Supplementary Figure 3E**; β = −0.30, 95% CI: −0.64 to 0.04, p = 0.08) and corticosterone (**Supplementary Figure 3E**; β = −0.27, 95% CI: −0.56 to 0.02, p = 0.07), and a suggestive increase in progesterone at Year 4 (**Supplementary Figure 3E**; β = 0.71, 95% CI: −0.09 to 1.51, p = 0.08).

### Baseline-Dependent ICS Effects and Metabolic Tracking

**Figure 2.**
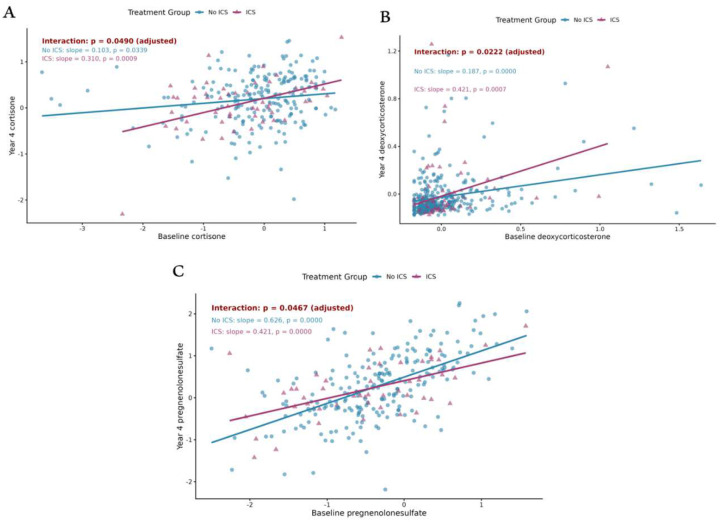
Baseline-endpoint relationships for metabolites with significant slope and ICS × baseline interactions. Scatter plots show individual baseline and Year 4 metabolite levels by treatment group. Regression lines represent unadjusted linear fits for visualization; interaction p-values are from ANCOVA models adjusted for age, race, and BMI. (A) Cortisone in females. (B) Deoxycorticosterone in males. (C) Pregnenolone sulfate in females.

As a follow-up analysis, we tested whether baseline-endpoint relationships differed by treatment group. For most metabolites (28/32 tests across sex), slopes were homogeneous (p > 0.05), satisfying the standard ANCOVA assumption of parallel regression lines; however, four metabolite-sex combinations showed significant interactions, including cortisone and pregnenolone sulfate in females; progesterone and deoxycorticosterone in males (**Supplementary Table 2**). For cortisone in females (Figure 2A) and deoxycorticosterone in males (Figure 2B), ICS users showed significantly stronger baseline-to-Year 4 tracking (slope = 0.31 and 0.42, respectively) than non-users (slope = 0.10 and 0.19, respectively). Conversely, for pregnenolone sulfate in females (Figure 2C), ICS users showed weaker tracking (slope = 0.42 vs. 0.63 in non-users). Although progesterone interaction in males was significant, neither individual slope reached significance, limiting interpretability and therefore not shown.

### Associations Between Steroid Metabolites and Clinical Outcomes

Given that at least one metabolite within the glucocorticoid, androgen, progestogen, and estrogen sub-pathways demonstrated significant or suggestive associations with ICS exposure in either the whole cohort or sex-stratified analyses, we focused our downstream clinical interpretation on these four biologically relevant pathways. In contrast, no metabolites within the mineralocorticoid sub-pathway showed significant associations with ICS exposure; therefore, they are not emphasized in the clinical outcome interpretation. We evaluated associations both longitudinally and at the Year-4 endpoint and present steroid sub-pathway-specific findings to facilitate biological interpretation.

### Glucocorticoid Pathway

**Figure 3.**
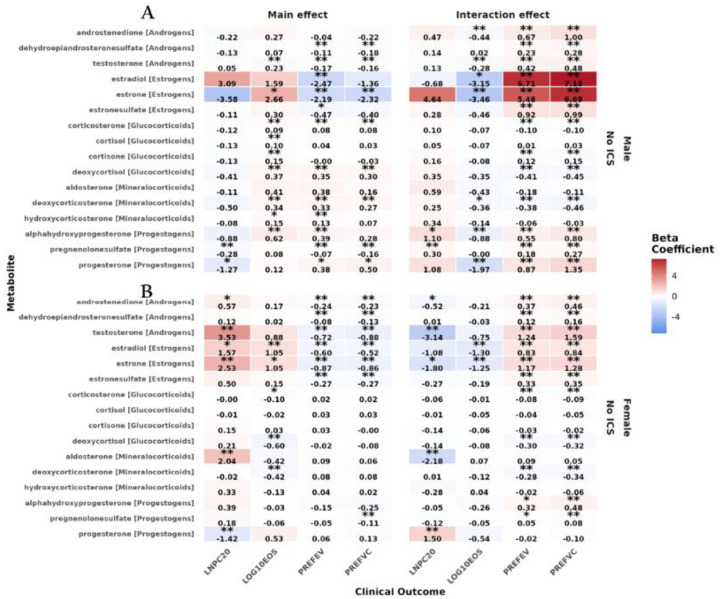

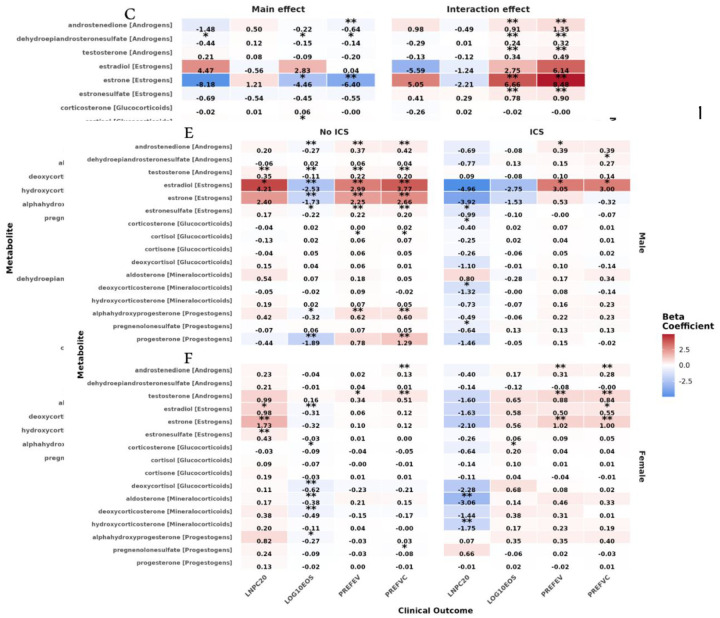
Associations between circulating steroid metabolites and clinical outcomes stratified by sex and ICS exposure. (A–D) Longitudinal associations estimated using mixed-effects models. (A) Main and interaction effects of metabolites on clinical outcomes in males not using ICS. (B) Main and interaction effects in females not using ICS. (C) Main and interaction effects in males using ICS. (D) Main and interaction effects in females using ICS. These models evaluate metabolite–time interactions representing associations between metabolite levels and changes in clinical outcomes over time. (E–F) Endpoint associations at Year 4 estimated using multivariable linear regression. (E) Associations between metabolites and clinical outcomes in males stratified by ICS exposure. (F) Associations in females stratified by ICS exposure. Main effect represents effect at preintervention. ** indicates p < 0.05; * indicates p < 0.10.

In male participants not receiving ICS, cortisone was significantly positively associated with longitudinal changes in FEV1 and FVC (Figure 3A), while precursor metabolites (11-deoxycortisol and corticosterone) showed significant negative associations with lung function in both sexes (Figure 3A & B). In ICS-treated participants (Figure 3C & D), these associations were attenuated, with sex-specific shifts and generally not statistically significant. Endpoint analyses at Year 4 were largely consistent with the longitudinal findings. Cortisol remained positively associated with lung function in non-ICS males (Figure 3E). In females, associations were more variable, with predominantly negative associations in non-ICS participants and positive, though non-significant, associations in ICS-treated participants (Figure 3F).

### Androgen Pathway

In both males and females who were not receiving ICS (Figure 3A & B), androgen metabolites (testosterone, DHEAS, and androstenedione) were positively associated with FEV1 and FVC trajectories. In males (non-ICS users), testosterone and androstenedione were negatively associated with eosinophil trajectories (Figure 3A). In ICS-treated males (Figure 3C), significant positive associations between androgens and lung function were largely preserved. In contrast, ICS-treated females showed attenuation of androgen–lung associations, which were largely inconsistent and not significant (Figure 3D). At Year 4, testosterone and androstenedione remained positively associated with lung function in males, regardless of treatment, and DHEAS demonstrated a significant negative association with PC_20_ in males treated with ICS (Figure 3E). In females, testosterone remained positively associated with lung function with or without ICS use, while androstenedione showed significantly stronger associations in ICS-treated participants (Figure 3F).

### Progestogen Pathway

In males not receiving ICS (Figure 3A), progesterone, 17α-hydroxyprogesterone (17-OHP), and pregnenolone sulfate showed significant positive associations with FEV_1_ and FVC trajectories. Also, progesterone and 17-OHP were negatively associated with eosinophil trajectories, and pregnenolone sulfate showed a significant positive association with log (PC_20_). In females not receiving ICS (Figure 3B), associations with lung function measures were more heterogeneous. Notably, progesterone showed a significant positive association with PC_20_. In ICS-treated males (Figure 3C), associations for pregnenolone sulfate, 17α-hydroxyprogesterone, and lung function were preserved, while progesterone associations were attenuated. In females receiving ICS (Figure 3D), associations were generally attenuated and non-significant. At Year 4, positive associations persisted in males (Figure 3E), particularly for 17α-hydroxyprogesterone and lung function. In females (Figure 3F), associations were variable and largely non-significant. However, 17-OHP demonstrated suggestive positive associations with FEV_1_ in ICS-treated females.

### Estrogen Pathway

In both sexes, not receiving ICS, estrone, estradiol, and estrone sulfate were significantly positively associated with lung function trajectories. In males not receiving ICS (Figure 3A), estrone showed a significant negative association with eosinophils longitudinally, and estradiol demonstrated a suggestive negative association over time. Amongst females not receiving ICS, estrone was significantly associated with lower eosinophils and suggestively associated with lower PC_20_ (Figure 3B). ICS exposure showed marked sex-specific modulation. In males using ICS (Figure 3C), estrone and estrone sulfate maintained significant positive associations with lung function. Associations between estrone, estradiol, and eosinophils were largely attenuated and non-significant. In females using ICS (Figure 3D), longitudinal estrogen–lung function associations were markedly attenuated, with predominantly non-significant and directionally inconsistent effects. At year 4 (Figure 3E & F), estrogen metabolites remained positively associated with lung function, regardless of ICS status, with several associations reaching statistical significance in males. In females using ICS, estrone showed significant positive associations with both FEV_1_ and FVC, and estradiol demonstrated a suggestive positive association with FVC. Immune phenotype analyses at Year 4 demonstrated a significant negative association between estradiol and eosinophils in non-ICS females. Also, estrone sulfate and estrone showed a significant positive association with PC_20_, indicating reduced airway hyperresponsiveness. In ICS-treated females, these immune associations were attenuated or directionally altered.

## Discussion

In this study, we evaluated the effects of ICS exposure on circulating steroid metabolites and their associations with clinical outcomes in children with asthma. While exogenous corticosteroids are known to suppress the hypothalamic–pituitary–adrenal (HPA) axis and lead to adrenal insufficiency (11), the extent to which these effects differ by sex at the metabolite level remains poorly understood. We first show that ICS exposure was associated with lower levels of endogenous glucocorticoids at the study endpoint, with consistent directional decreases observed in longitudinal analyses across the full cohort, suggesting that chronic ICS use is associated with suppression of systemic glucocorticoid metabolism, which is consistent with previous studies (11, 13). In contrast, selected upstream steroid precursors, including 17α-hydroxyprogesterone and testosterone, showed relative increases over time, consistent with previously reported inverse relationships between cortisol and upstream steroid intermediates (22), and suggesting altered steroidogenic flux under ICS exposure. We next observed marked sex-specific differences in ICS–metabolite associations. Males receiving ICS demonstrated greater reductions in glucocorticoids at follow-up, whereas females showed more stable or modestly increasing trajectories, resulting in attenuated differences at the endpoint. Interestingly, a prior study has reported a higher proportion of clinical responders to ICS among females (23), consistent with our observation of less pronounced glucocorticoid suppression in females than in males. Also, the heterogeneous slope patterns we observed reveal that ICS treatment does not uniformly affect all steroid pathways. For glucocorticoids (cortisone) in females and mineralocorticoid precursors (deoxycorticosterone) in males, ICS strengthened baseline-endpoint tracking, suggesting that exogenous glucocorticoid exposure preserves endogenous steroid set-points. In contrast, in females, ICS weakened tracking of progestogen precursors (pregnenolone sulfate), indicating pathway-specific suppression that drives convergence toward a common metabolic state. These findings suggest that the metabolic effects of ICS exposure may differ by sex during childhood and adolescence. Further, our pubertal analysis results indicate that ICS use may differentially influence steroid metabolite dynamics across sex and pubertal stage, although interpretation is limited by sample size and should be considered exploratory.

Next, to determine whether endogenous steroid metabolites serve as biomarkers of clinical outcomes and whether ICS exposure modifies these relationships, we examined associations between metabolite levels and lung function (FEV_1_ and FVC), eosinophil counts, and airway hyperresponsiveness, stratified by sex and treatment status. Precursors in the glucocorticoid sub-pathway were negatively associated with lung function, whereas mature active glucocorticoids showed positive associations in non-ICS users, suggesting that precursor accumulation may reflect poorer respiratory mechanics across sexes in the absence of ICS exposure. In ICS-treated males, these endogenous glucocorticoid–lung function relationships appeared attenuated, with longitudinal associations becoming more uniformly negative and non-significant. This pattern is consistent with the possibility that exogenous glucocorticoid exposure may alter the endogenous steroid–lung function relationships observed in untreated males. In contrast, females receiving ICS showed a shift toward more positive trajectory associations, although these were not statistically significant; the consistent directional patterns suggest potential sex-specific differences in response to exogenous glucocorticoid exposure. While prior pediatric studies have linked higher levels of glucocorticoid precursors relative to cortisol with adverse lung outcomes (24), broadly consistent with the directional patterns observed in our data, the extent to which ICS exposure influences these relationships remains underexplored. Our findings extend this framework by suggesting that these associations may vary under ICS treatment and could differ by sex. Further, androgens demonstrated robust positive associations with lung function and inverse associations with eosinophils, consistent across sexes but more pronounced in males. Progestogen and estrogen pathways also showed positive associations with lung function, although these relationships were more heterogeneous in females. ICS exposure modified metabolite–clinical outcome associations in a pathway- and sex-specific manner. In general, ICS attenuated longitudinal associations between endogenous steroids and clinical outcomes, particularly in females, suggesting disruption of endogenous steroid physiology. However, several associations, particularly androgen–lung function relationships in males and estrogen–lung function relationships at the endpoint, were either preserved or re-emerged, indicating partial adaptation over time. Previous studies have shown that higher androgen levels are associated with improved lung function, reduced airway inflammation, and lower FeNO in asthma (25). Estrogen has been linked to enhanced type 2 airway inflammation, and progesterone shows more variable effects on airway physiology and asthma severity (26), which may contribute to the heterogeneous associations observed in our data. Taken together, these findings suggest that ICS therapy may influence systemic steroid metabolism and alter the relationship between endogenous steroid pathways and respiratory outcomes. The observed sex-specific differences highlight the importance of considering biological sex in studies of steroid metabolism and asthma treatment. Additionally, the persistence of associations for specific metabolites, particularly steroid precursors, suggests their potential utility as biomarkers of disease progression or treatment response.

A key limitation of this study is the presence of baseline metabolic differences between participants randomized to the ICS and control groups prior to intervention. To account for potential confounding, baseline metabolite levels were included as covariates in the mixed models. The limited attenuation of effect sizes following this adjustment suggests that baseline differences alone do not fully explain the observed longitudinal associations. However, unmeasured clinical factors, such as asthma severity or systemic inflammation, may still contribute to the reduced steroid levels observed among participants receiving ICS. In addition, the small sample sizes in the pre- and post-puberty stratified analyses may limit the stability of model estimates and reduce power to detect moderate effects. Further studies integrating mechanistic and longitudinal data will be important to clarify the biological pathways underlying these observations and to determine their clinical implications.

## Conclusion

In summary, our study demonstrates that inhaled corticosteroid exposure is associated with sustained alterations in steroid biosynthesis pathways in children with asthma, with clear evidence of sex-specific effects on both metabolite profiles and their relationships with clinical outcomes. The observed suppression of endogenous glucocorticoids alongside compensatory shifts in upstream steroid precursors suggests disruption of steroidogenic flux under chronic ICS use. Importantly, the differential modulation of metabolite–clinical outcome associations by sex highlights the need to consider biological sex in both mechanistic studies and therapeutic strategies. These findings support the potential utility of targeted steroid metabolomics as a tool for monitoring treatment effects and identifying biomarkers of disease progression and response. Future studies in larger and more diverse populations, with deeper integration of pubertal and hormonal data, are warranted to validate these observations and to inform precision medicine approaches in asthma management.

## Supplementary Material

Supplementary Files

This is a list of supplementary files associated with this preprint. Click to download.

- SupplementaryFile.docx

## Figures and Tables

**Table 1.** Participants’ Characteristics by Treatment Status

Group	Variable	Overall	Non-ICS	ICS
Baseline	N	664	527	137
	Age, mean ± SD (years)	8.9 ± 2.1	8.8 ± 2.1	9.3 ± 2.1
	BMI, mean ± SD (kg/m^2^)	18.3 ± 3.5	18.1 ± 3.4	18.9 ± 3.9
	Sex, n (%)	Male: 403 (60.7%), Female: 261 (39.3%)	Male: 317 (60.2%), Female: 210 (39.8%)	Male: 86 (62.8%), Female: 51 (37.2%)
	Race/Ethnicity, n (%)	African American: 90 (13.6%); Hispanic: 74 (11.1%); Other: 72 (10.8%); White: 428 (64.5%)	African American: 67 (12.7%); Hispanic: 46 (8.7%); Other: 51 (9.7%); White: 363 (68.9%)	African American: 23 (16.8%); Hispanic: 28 (20.4%); Other: 21 (15.3%); White: 65 (47.4%)
Year 4	N	664	527	137
	Age, mean ± SD (years)	12.9 ± 2.1	12.8 ± 2.1	13.3 ± 2.1
	BMI, mean ± SD (kg/m^2^)	21.6 ± 4.8	21.4 ± 4.7	22.2 ± 5.0
	Sex, n (%)	Male: 403 (60.7%), Female: 261 (39.3%)	Male: 317 (60.2%), Female: 210 (39.8%)	Male: 86 (62.8%), Female: 51 (37.2%)
	Race/Ethnicity, n (%)	African American: 90 (13.6%); Hispanic: 74 (11.1%); Other: 72 (10.8%); White: 428 (64.5%)	African American: 67 (12.7%); Hispanic: 46 (8.7%); Other: 51 (9.7%); White: 363 (68.9%)	African American: 23 (16.8%); Hispanic: 28 (20.4%); Other: 21 (15.3%); White: 65 (47.4%)

## Data Availability

Main data, model outputs and detailed preprocessing and data analysis parameters are provided within the manuscript, methods or supplementary information files. Requests for other data and materials will be reviewed by the cohort and contact PI for targeted metabolomics data Dr. Jessica Lasky-Su at rejas@channing.harvard.edu, subject to appropriate execution of a Material Transfer or Data Use Agreement and verification of appropriate IRB/ethics approvals and institutional compliance. All custom R code used for data processing and statistical analyses can be made openly available on GitHub with request to Dr. Kachroo at pk784@shp.rutgers.edu.

## List of abbreviations

Pre-FEV_1_: pre-bronchodilator forced expiratory volume in 1 second
Pre-FVC: pre-bronchodilator forced vital capacity
LNPC20: natural logarithm of the provocative concentration 20%
EOS: eosinophil
ICS: inhaled corticosteroid
BMI: body mass index
CAMP: childhood asthma management program
CI: confidence interval

## References

[1] Swed S., Sawaf B., Al-Obeidat F., Hafez W., Rakab A., Alibrahim H., … & Cherrez-Ojeda I. (2024). Asthma prevalence among United States population insights from NHANES data analysis. Scientific Reports, 14(1), 8059. 38580691 10.1038/s41598-024-58429-5 PMC10997649

[2] Schatz M., & Camargo C. A. Jr (2003). The relationship of sex to asthma prevalence, health care utilization, and medications in a large managed care organization. Annals of allergy, asthma & immunology, 91(6), 553–558. 10.1016/S1081-1206(10)61533-5 14700439

[3] Clausen J. L., Coates A. L., & Quanjer P. H. (1997). Measurement of lung volumes in humans: review and recommendations from an ATS/ERS workshop. European Respiratory Journal, 10(6), 1205–1206. 9192917 10.1183/09031936.97.10061205

[4] Stocks J., & Quanjer P. H. (1995). Reference values for residual volume, functional residual capacity and total lung capacity. ATS Workshop on Lung Volume Measurements. Official Statement of The European Respiratory Society. European Respiratory Journal, 8(3), 492–506. 7789503 10.1183/09031936.95.08030492

[5] Borish L., Chipps B., Deniz Y., Gujrathi S., Zheng B., Dolan C. M., & TENOR Study Group. (2005). Total serum IgE levels in a large cohort of patients with severe or difficult-to-treat asthma. Annals of Allergy, Asthma & Immunology, 95(3), 247–253. 10.1016/S1081-1206(10)61221-5 16200815

[6] Sears M. R., Burrows B., Herbison G. P., Flannery E. M., & Holdaway M. D. (1993). Atopy in childhood. III. Relationship with pulmonary function and airway responsiveness. Clinical & Experimental Allergy, 23(11), 957–963. 10779284 10.1111/j.1365-2222.1993.tb00281.x

[7] Becklake M. R., & Kauffmann F. (1999). Gender differences in airway behaviour over the human life span. Thorax, 54(12), 1119–1138. 10567633 10.1136/thx.54.12.1119 PMC1763756

[8] Fuseini H., & Newcomb D. C. (2017). Mechanisms driving gender differences in asthma. Current allergy and asthma reports, 17, 1–9. 28332107 10.1007/s11882-017-0686-1 PMC5629917

[9] DeBoer M. D., Phillips B. R., Mauger D. T., Zein J., Erzurum S. C., Fitzpatrick A. M., … & Gerald Teague W. (2018). Effects of endogenous sex hormones on lung function and symptom control in adolescents with asthma. BMC pulmonary medicine, 18, 1–10. 29631584 10.1186/s12890-018-0612-x PMC5891903

[10] Kearns N., Maijers I., Harper J., Beasley R., & Weatherall M. (2020). Inhaled corticosteroids in acute asthma: a systemic review and meta-analysis. The Journal of Allergy and Clinical Immunology: In Practice, 8(2), 605–617. 31521830 10.1016/j.jaip.2019.08.051

[11] Gurnell M., Heaney L. G., Price D., & Menzies-Gow A. (2021). Long-term corticosteroid use, adrenal insufficiency and the need for steroid-sparing treatment in adult severe asthma. Journal of internal medicine, 290(2), 240–256. 33598993 10.1111/joim.13273 PMC8360169

[12] Joshi A. D., Rahnavard A., Kachroo P., Mendez K. M., Lawrence W., Julián-Serrano S., … & Darst B. F. (2023). An epidemiological introduction to human metabolomic investigations. Trends in Endocrinology & Metabolism, 34(9), 505–525. 37468430 10.1016/j.tem.2023.06.006 PMC10527234

[13] Kachroo P., Stewart I. D., Kelly R. S., Stav M., Mendez K., Dahlin A., … & Lasky-Su J. A. (2022). Metabolomic profiling reveals extensive adrenal suppression due to inhaled corticosteroid therapy in asthma. Nature medicine, 28(4), 814–822. 10.1038/s41591-022-01714-5 PMC9350737 35314841

[14] Tran D. T., Chen Y., Ramirez L. G., Lasky-Su J. A., Wu A. C., Tantisira K. G., … & Dahlin A. (2025). Plasma pharmacometabolomics of inhaled corticosteroid–related adrenal suppression in asthma. Journal of Allergy and Clinical Immunology, 155(6), 1857–1865. 40089116 10.1016/j.jaci.2025.02.037

[15] Chen Y., Checa A., Zhang P., Huang M., Kelly R. S., Kim M., … & Lasky-Su J. A. (2024). Sphingolipid classes and the interrelationship with pediatric asthma and asthma risk factors. Allergy, 79(2), 404–418. 38014461 10.1111/all.15942 PMC11175620

[16] Mendez K. M., Kachroo P., Prince N., Huang M., Cote M., Chu S. H., … & Lasky-Su J. A. (2025). Exploring the varied clinical presentation of pediatric asthma through the metabolome. American Journal of Respiratory and Critical Care Medicine, 211(5), 737–748. 39965055 10.1164/rccm.202407-1382OC

[17] Chen Y., Zhang P., Huang M., Kachroo P., Checa A., Chen Q., … & Lasky-Su J. A. (2026). The ratio of circulatory levels of sphingolipids to steroids predicts asthma exacerbations. Nature Communications, 17(1), 545. 10.1038/s41467-025-67436-7 PMC12815949 41554715

[18] Childhood Asthma Management Program Research Group. (1999). The childhood asthma management program (CAMP): design, rationale, and methods. Controlled clinical trials, 20(1), 91–120. 10027502

[19] Childhood Asthma Management Program Research Group. (2000). Long-term effects of budesonide or nedocromil in children with asthma. New England Journal of Medicine, 343(15), 1054–1063. 11027739 10.1056/NEJM200010123431501

[20] Strunk R. C., Sternberg A. L., Szefler S. J., Zeiger R. S., Bender B., Tonascia J., & Childhood Asthma Management Program (CAMP) Research Group. (2009). Long-term budesonide or nedocromil treatment, once discontinued, does not alter the course of mild to moderate asthma in children and adolescents. The Journal of pediatrics, 154(5), 682–687. 19167726 10.1016/j.jpeds.2008.11.036 PMC2942076

[21] Team R. C. (2020). RA language and environment for statistical computing, R Foundation for Statistical. Computing.

[22] Charmandari E., Matthews D. R., Johnston A., Brook C. G., & Hindmarsh P. C. (2001). Serum cortisol and 17-hydroxyprogesterone interrelation in classic 21-hydroxylase deficiency: is current replacement therapy satisfactory?. The Journal of Clinical Endocrinology & Metabolism, 86(10), 4679–4685. 11600525 10.1210/jcem.86.10.7972

[23] Wu Y. F., Su M. W., Chiang B. L., Yang Y. H., Tsai C. H., & Lee Y. L. (2017). A simple prediction tool for inhaled corticosteroid response in asthmatic children. BMC pulmonary medicine, 17(1), 176. 29216859 10.1186/s12890-017-0533-0 PMC5721661

[24] Watterberg K. L., Gerdes J. S., & Cook K. L. (2001). Impaired glucocorticoid synthesis in premature infants developing chronic lung disease. Pediatric Research, 50(2), 190–195. 11477202 10.1203/00006450-200108000-00005

[25] Zein J. G., McManus J. M., Sharifi N., Erzurum S. C., Marozkina N., Lahm T., … & Gaston B. (2021). Benefits of airway androgen receptor expression in human asthma. American journal of respiratory and critical care medicine, 204(3), 285–293. 33779531 10.1164/rccm.202009-3720OC PMC8513596

[26] Borrelli R., Brussino L., Lo Sardo L., Quinternetto A., Vitali I., Bagnasco D., … & Nicola S. (2025). Sex-Based differences in asthma: Pathophysiology, hormonal Influence, and genetic mechanisms. International Journal of Molecular Sciences, 26(11), 5288. 40508095 10.3390/ijms26115288 PMC12154264

